# Prodrugs for Improved Drug Delivery: Lessons Learned from Recently Developed and Marketed Products

**DOI:** 10.3390/pharmaceutics12111031

**Published:** 2020-10-29

**Authors:** Milica Markovic, Shimon Ben-Shabat, Arik Dahan

**Affiliations:** Department of Clinical Pharmacology, School of Pharmacy, Faculty of Health Sciences, Ben-Gurion University of the Negev, Beer-Sheva 84105, Israel; milica@post.bgu.ac.il (M.M.); sbs@bgu.ac.il (S.B.-S.)

**Keywords:** prodrugs, biopharmaceutics, drug absorption, oral administration, drug targeting, drug delivery, ProTide

## Abstract

Prodrugs are bioreversible, inactive drug derivatives, which have the ability to convert into a parent drug in the body. In the past, prodrugs were used as a last option; however, nowadays, prodrugs are considered already in the early stages of drug development. Optimal prodrug needs to have effective absorption, distribution, metabolism, and elimination (ADME) features to be chemically stable, to be selective towards the particular site in the body, and to have appropriate safety. Traditional prodrug approach aims to improve physicochemical/biopharmaceutical drug properties; modern prodrugs also include cellular and molecular parameters to accomplish desired drug effect and site-specificity. Here, we present recently investigated prodrugs, their pharmaceutical and clinical advantages, and challenges facing the overall prodrug development. Given examples illustrate that prodrugs can accomplish appropriate solubility, increase permeability, provide site-specific targeting (i.e., to organs, tissues, enzymes, or transporters), overcome rapid drug metabolism, decrease toxicity, or provide better patient compliance, all with the aim to provide optimal drug therapy and outcome. Overall, the prodrug approach is a powerful tool to decrease the time/costs of developing new drug entities and improve overall drug therapy.

## 1. Introduction

Powerful modern drug discovery techniques, such as combinatorial chemistry and high-throughput screening, enable the discovery of novel chemical entities with high pharmacological efficacy [1]. These techniques have also created a new challenge, as many of the new drug candidates have unfavorable physicochemical features and still require chemical modification or use of formulation technologies to achieve adequate performance and pass the rigorous drug development process successfully [2]. Chemical modifications may range from creating salts for improved dissolution rate [3] to increasing lipophilicity, e.g., through incorporating alkyl moieties into the drug molecule; however, these modifications may result in the loss/reduction of pharmacological activity compared to the parent drug [4]. Various formulation technologies can be employed to overcome physicochemical obstacles [5,6,7], though they may or may not be successful in reaching adequate drug delivery. The prodrug approach represents a potentially effective solution to these obstacles [8].

Prodrugs are bioreversible, inactive drug derivatives, which have the ability to convert into the active parent drug within the human body. The prodrug approach is used to overcome biopharmaceutic, pharmacokinetic, or pharmacodynamic obstacles, including poor chemical stability, solubility limitations, lack of site-specificity, extensive drug metabolism, passing through biological barriers, exploiting endogenous metabolic pathways, toxicity, or compliance obstacles (unacceptable taste/odor), all in favor of optimal oral bioavailability and consequent therapeutic effect [9]. The prodrug approach is used for the optimization of newly discovered chemical entities, as well as to improve the properties of already marketed drugs. The United States Food and Drug Administration (FDA) have approved around 30 prodrugs in the past decade (12% of all approved new small-molecule entities). Approximately 10% of all commercially available medicines in the world are considered to be prodrugs [10].

In the past, the prodrug approach used to be considered as a last resort in drug development; not only is this no longer the case but nowadays, the prodrug approach is considered at very initial stages of drug research and development. Making a prodrug indeed means dealing with a new chemical entity; however, prodrugs do not come with the same cost as developing a novel drug. The improved performance (compared to the parent drug) expedites the process of drug development, which eventually may save time, money, and efforts [9,11,12,13].

We can distinguish between the traditional and modern approaches to prodrug design. The traditional prodrug approach includes covalent binding of the parent drug to either hydrophilic functional groups (e.g., phosphate, sulfate) in order to improve the solubility of the parent drug [14] or lipophilic groups (e.g., alkyl, aryl) in order to improve passive permeability [15]. In this strategy, it is difficult to direct the prodrug towards a specific site; nevertheless, this is an important tool to improve physicochemical and/or biopharmaceutical properties and overcome obstacles that a drug faces in the body. The potential aims of this approach include to modify drug pharmacokinetics, accomplish extended drug release, decrease toxicity, and more [11]. On the other hand, the molecular revolution in biology and medicine has allowed a modern approach to prodrug design, which includes molecular/cellular parameters (membrane influx/efflux transporters and cellular protein expression and distribution) and is directed towards specific target molecules in the body [11,16,17,18]. The carrier is covalently attached to the parent drug in order to selectively target certain enzymes/transporters. The opportunity to control the mechanism and site of parent drug release from the prodrug, which, in turn, may enable targeting the free drug to the site(s) it is actually needed, presents a significant advantage for the modern prodrug approach and a challenge for the prodrug development; this step can also be optimized through powerful computational simulations [19,20,21].

**Figure 1:**
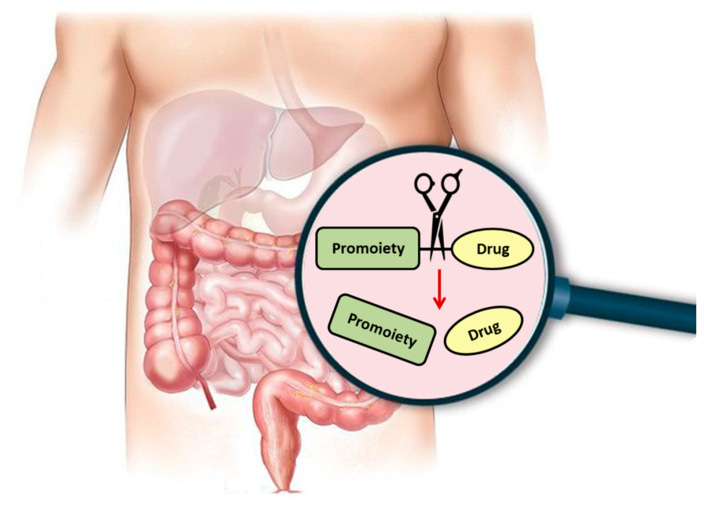
Illustration of activation of orally administered prodrugs in the gastrointestinal tract. Scissors represent chemical/enzymatic activation of the carrier-drug with consequent release of the reaction products: carrier and drug alone.

Regardless of the specific approach or purpose, following prodrug intake, it needs to undergo an activation step in which the free parent drug is liberated, and the pharmacological effect can then be exerted (Figure 1). The prodrug activation can be either chemical or enzyme-mediated conversion to the parent drug. This step is unique for prodrugs and distinguishes their development from regular drugs. It is likely that this extra step has contributed to the past reluctance of the pharmaceutical industry to take a prodrug approach early on in the development process; nowadays, it is well recognized that this extra step also brings great potential with it. Numerous prodrug advantages and the number of registered prodrugs in the last decades demonstrate how invaluable this approach has grown to be.

Prodrugs can be developed for many routes of administration: oral, intravenous, intramuscular, inhalation, transdermal, and more [22]. Nevertheless, the oral route is the most common and desirable route of administration; therefore, oral prodrugs are emphasized in this article. Our main goals are to provide the key concepts of the prodrug approach, provide an overview of successfully registered oral prodrugs, and analyze their gained therapeutic advances in comparison to the parent drug. Furthermore, successful examples of prodrugs, either already on the market or in clinical trials, are presented, emphasizing their pharmaceutical/therapeutic/clinical advantages compared to the parent drug.

## 2. Prodrug Activation

As mentioned above, one of the main steps to consider in prodrug design is the activation mechanism, which liberates the active parent drug from the prodrug in an effective and well-characterized manner, in order to meet the therapeutic purpose. Prodrug activation can be based on chemical processes (e.g., oxido-reduction) or can occur via enzyme-mediated hydrolysis, from oxidoreductases (i.e., cytochrome P450), hydrolytic enzymes (i.e., carboxylesterases, phosphatase, esterase) to transferases and lyases [23].

In oral prodrug delivery, significant challenges are the numerous absorption barriers and consecutive sites of potential metabolism on the way to the systemic blood circulation. A key issue with oral prodrugs is whether the prodrug will be activated before or after absorption and reaching the systemic circulation. Here, we present two examples of each approach and the importance of choosing the pre/post-absorption activation approach.

**Figure 2:**
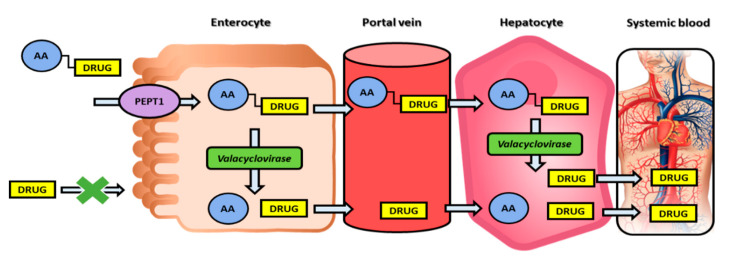
Double-targeted prodrug strategy illustration: binding to the PEPT1 transporter and activation through enzyme valacyclovirase. PEPT1, peptide transporter 1; AA, amino acid.

The post-absorption activation approach is demonstrated on the nucleoside analogs, in which case the optimal prodrug is stable during transport/delivery but is freely cleaved to liberate the parent drug once at the target site. Valacyclovir, the 5′-valyl ester prodrug of the anti-herpetic drug acyclovir (Table 1), demonstrates 3–5 fold higher systemic bioavailability when compared to the parent drug. This increase in the prodrug bioavailability is due to the human peptide transporter 1 (hPEPT1), which mediates its intestinal absorption [24]. Nevertheless, the efficacy of valacyclovir predominantly relies on fast transformation to the acyclovir in-vivo; it has been shown that valacyclovir is stable in the gastrointestinal lumen (which allows its hPEPT1-mediated transport and increased absorption), followed by a high predisposition for intracellular enzymatic-mediated activation [24]. Following the discovery of valacyclovir, the enzyme responsible for this bioconversion to acyclovir has been identified, and it has been shown that this enzyme is a serine hydrolase, consequently named human valacyclovirase [25]. It was later shown that human valacyclovirase is an optimal targeting molecule for α-amino acid ester prodrugs, whereas it is mildly active toward amide prodrugs [26,27]. Subsequent acyclovir liberation from valacyclovir allows the free drug to be selectively taken by the infected cells and followed by acyclovir triphosphorylation catalyzed by host cell enzymes, resulting in acyclovir triphosphate, which prevents viral DNA synthesis by inhibiting the viral DNA polymerase [28]. This example includes both transporter-mediated transport for increasing acyclovir intestinal absorption and a specific prodrug-activating enzyme, making it a ‘double-targeted’ approach [24]; this is an inspiring example for a promising way to exploit modern molecular knowledge in the development of novel prodrugs and improving overall drug targeting and therapy (Figure 2).

A novel phospholipid-based prodrug approach has been developed and studied in our research group [29,30,31,32,33]. Phospholipids (PL) are natural substrates of an enzyme phospholipase A_2_ (PLA_2_), which is responsible for the hydrolysis of the *sn*-2 fatty acyl bond of the PL, thereby liberating a free fatty acid (FA) and a lysophospholipid [34]. PLA_2_ has been reported to be significantly overexpressed in the inflamed intestinal patched of inflammatory bowel disease (IBD) patients [35,36], likely due to its important role in the pathogenesis of many inflammatory/cancer processes [37]. Our prodrug design includes a drug conjugated to the *sn*-2 position of the PL in place of a FA [38]. Previous reports have suggested that the PLA_2_ enzymes don’t have any specific FA selectivity [39], signifying that carefully designed PL-drug conjugates may be specifically activated by this enzyme. Hence, this PL-based prodrug design may exploit PLA_2_ catalytic action, thereby liberating the free drug moiety from the PL-drug complex. Since the intact complex cannot be absorbed and owing to the enzyme overexpression at the inflamed intestine, oral administration of this prodrug may target the free drug specifically to the diseased intestinal patches, representing a significant therapeutic advance to our treatment of IBD patients [30,31,32,33]. This example illustrates beneficial prodrug activation already within the intestinal lumen, prior to absorption.

Another exciting approach to prodrug targeting to specific tissues is the directed enzyme prodrug therapy (DEPT), which uses enzymes artificially brought into the body to activate the prodrug and release the active moiety. This strategy has been shown for antibodies (ADEPT), genes (GDEPT, also known as suicide gene therapy, where the enzyme is created inside the target cell, by means of a gene supplied by gene therapy), viruses (VDEPT, where viruses carry the gene for GDEPT), polymer-directed enzyme prodrug therapy (PDEPT), and clostridia-directed enzyme prodrug therapy (CDEPT), which takes advantage of the hypoxic cancer surroundings to target drugs to tumors through a bacteria residing in the cancer tissues [40].

## 3. Clinical Prodrugs for Oral Drug Delivery

In this section, we discuss prominent examples of successful prodrugs for oral administration registered by the FDA in the past decade, emphasizing the improvements accomplished by developing the prodrug as compared to the free drug. A key setback when it comes to oral drugs is inadequate intestinal permeability and, consequently, absorption due to low drug lipophilicity [41,42]. Several prodrugs have been developed to overcome this obstacle, such as direct thrombin inhibitor dabigatran etexilate [43] and telotristat etiprate for carcinoid syndrome diarrhea [44] and fingolimod hydrochloride [45] and dimethyl fumarate [46] for the therapy of multiple sclerosis.

An interesting example of increased permeability (through increasing lipophilicity), as well as prodrug targeting, is demonstrated in recently approved antiviral drugs—tenofovir and sofosbuvir. Both prodrugs have been developed using a novel ProTide technology. The ProTide technology allows nucleotide analog delivery to the cells by masking the monophosphate/monophosphonate hydroxyl groups by an aromatic group of amino acid ester [47]. The prodrug, tenofovir alafenamide hemifumarate, developed via this technology, was approved by the FDA in 2015 as a tablet, a fixed-dose mixture of elvitegravir, cobicistat, and emtricitabine; it was also the first clinically efficient nucleoside analog produced with this technology (Table 1). As opposed to previously used tenofovir disoproxil, which was cleaved both intracellularly and extracellularly, tenofovir alafenamide has been shown to be cleaved largely intracellularly, allowing optimal tissue targeting with lower doses (which has helped to reduce kidney toxicity and bone marrow depletion). The following year, tenofovir alafenamide was approved as a drug for hepatitis B virus (HBV) infection. ProTide approach has also been used for the prodrug sofosbuvir, an antiviral (highly potent inhibitor of hepatitis C virus NS5B polymerase) nucleotide analog, in order to improve permeability and liver targeting [62].

One of the recent prominent examples of marketed prodrugs is the baloxavir marboxil, approved by the FDA in 2018. Baloxavir marboxil is an antiviral drug for influenza, the first in nearly 20 years (Table 1) [59]. Due to reduced susceptibility or resistance of neuraminidase inhibitors (oseltamivir, zanamivr, and peramivir) to the influenza viruses, there has been a strong need for an alternative with a different mechanism of action. Baloxavir marboxil is a prodrug of baloxavir acid whose mechanism of action is selective inhibition of influenza cap-dependent endonuclease. Clinical trials have demonstrated a bigger decrease in viral load with baloxavir marboxil vs. oseltamivir [58,59]. In addition, combination with neuraminidase inhibitors may lead to relieving concerns about the development of resistance [63].

One of the other reasons for using a prodrug approach is enhancing drug aqueous solubility. An interesting example of a novel approach for prodrug solubility improvement is isavuconazonium sulfate (Table 1), an acyloxyalkyl triazolium salt of the antifungal agent, isavuconazole. The primary goal was to improve solubility for parenteral use; however, the exceptional improvement in solubility has allowed the development of both parenteral and oral prodrug administration. Isavuconazonium is entirely cleaved by esterases (mainly butyrylcholinesterase) in the plasma following parenteral use; following oral administration, quick presystemic/systemic metabolism in the gut, liver, and plasma converts the prodrug to isavuconazole, with almost complete bioavailability following both routes of administration (~98%) [49]. Isavuconazonium sulfate was approved by the FDA as a drug for invasive aspergillosis and invasive mucormycosis in adults. Another prodrug with improved solubility registered by the FDA in the past decade is tedizolid phosphate for acute skin infections [48].

The advantage of the modern targeted prodrug approach is demonstrated for gabapentin enacarbil, a prodrug approved by the FDA in 2011 for restless leg syndrome/postherpetic neuralgia (Table 1). Gabapentin enacarbil is a prodrug of gabapentin and a substrate for two high-capacity nutrient transporters—MCT-1 and sodium-dependent multivitamin transporter (SMVT)—with a wide distribution throughout the human gastrointestinal tract [50]. The prodrug design has increased the capacity of transport and allowed the delivery of higher gabapentin doses in appropriate dose proportion, circumventing uptake saturation at clinical doses.

An example of prodrug utilization for prolonged drug action, an N-acylsulfonamide prodrug of prostacyclin agonist, ACT-333679, selexipag, was approved by the FDA for pulmonary arterial hypertension (Table 1) [51]. It was approved as an agonist of the prostacyclin receptor, which leads to vasodilatation in the pulmonary circulation. ACT-333679 is up to 37-fold more potent than selexipag, and it exhibits 3–4 fold higher levels of the free drug following oral administration in humans, making the ACT-333679 the main contributor of the selexipag efficacy [64]. Selexipag was developed in order to prolong the drug effect duration, which has been accomplished by slow bioconversion via hepatic carboxylesterase 1, and it has improved peak-to-trough fluctuation (dose-proportional pharmacokinetic exposure), as well as decreased side effects [51].

Some prodrugs are designed in order to improve the pharmacokinetic profile for the parent drug. Examples include fesoterodine fumarate, used for the treatment of overactive bladder syndrome, was developed in order to decrease interpatient pharmacokinetic variability [65]; angiotensin II antagonist azilsartan medoxomil monopotassium, which allows higher bioavailability, characterized by larger ability to control 24-h systolic blood pressure, in comparison to other sartans [38]; valbenazine tosylate for tardive dyskinesia has improved pharmacokinetic profile [66].

Numerous prodrugs have been approved to accomplish specific goals; bioprecursor prodrug, a blood thinner, and inhibitor of the platelet P2Y_12_ prasugrel hydrochloride through metabolic activation have accomplished sulfenylation of the platelet P2Y_12_ receptor, thus exhibiting its pharmacological effect (Table 1) [52]; eslicarbazepine acetate, the anticonvulsant has demonstrated improved safety profile and reduced drug interactions [67]; droxidopa, used for neurogenic orthostatic hypotension/off-label Parkinson disease, has demonstrated enhanced brain permeability [68].

**Figure 3:**
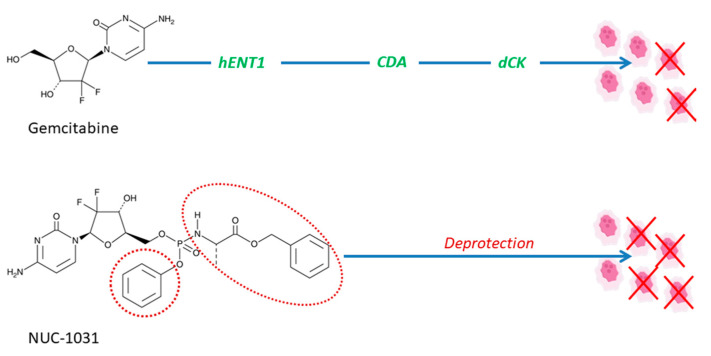
NUC-1031 overcomes the main resistance mechanisms associated with gemcitabine, in particular, transport via human equilibrative nucleoside transporters (hENT1), breakdown via cytidine deaminase (CDA), and activation via deoxycytidine kinase (dCK). NUC-1031 is designed to create and retain higher drug levels concentrations of metabolite inside the tumor in comparison to gemcitabine. The promoieties are shown with a dashed red line.

At the moment, there are numerous clinical trials being conducted to evaluate the efficacy of prodrugs for different therapeutic purposes; here, we present some interesting examples. For instance, a trial is underway to evaluate the efficacy of anticancer prodrug NUC-1031, produced via ProTide technology, in patients with advanced biliary tract cancer (Table 1) [53]. NUC1031 is formed as a pre-activated monophosphate form of gemcitabine, which, due to the protective phosphoramidate transformation, has the ability to overcome resistance mechanisms of gemcitabine, including resistance to human equilibrative nucleoside transporters-based, nucleoside activation resistance (deoxycytidine kinase deficiency), and pyrimidine deamination (via deoxycytidine deaminase), presented in Figure 3 [54].

An ongoing trial is conducted for the AKR1C3-activated prodrug OBI-3424 for patients with relapsed/refractory T-cell acute lymphoblastic leukemia (Table 1) [55]; OBI-3424 also entered trials for hepatocellular carcinoma and castrate-resistant prostate cancer [56]. OBI-3424 selectively releases a potent DNA alkylating agent in the presence of the aldo-keto reductase family 1 member C3 (AKR1C3), overexpressed in cancerous tissues; it is distinguished from traditional alkylating agents (i.e., cyclophosphamide, ifosfamide) by its selective mode of activation [57]. Enalapril, a well-known model angiotensin-converting enzyme inhibitor (ACEI) prodrug, activated by carboxylesterase 1, is currently under clinical investigation in order to determine the influence of genetic variability of the enzyme on the enalapril activation, pharmacokinetics, and pharmacodynamics in a multiple-dose healthy volunteer setting. The conclusions from this study could be a major stepping stone for a more personalized use of ACEI prodrugs [69].

Remdesivir is an antiviral nucleotide prodrug (ProTide) [70], which inhibits viral RNA synthesis [61]. Nucleoside analogs have poor permeability through the cells; therefore, modifications, such as monophosphate, ester, or, in this case, phosphoramidate prodrug, can significantly improve drug permeability through cells and metabolism, leading to the liberation of the free nucleoside or phosphorylated nucleoside within the cells [71]. Remdesivir was developed as a therapeutic agent for treating RNA-based viruses threatening to become a global pandemic, including Ebola virus (EBOV) and the *Coronaviridae* family of viruses. It has been evaluated in humans against acute Ebola virus disease [72], and on May 1st, 2020, the Food and Drug Administration (FDA) gave out an Emergency Use Authorization (EUA) for the emergency use of remdesivir for treating hospitalized patients with severe 2019 coronavirus disease (COVID-19) (Table 1) [60]. Remdesivir is still an investigational drug and, as of yet, has not been approved for any indication.

Despite the evident success in the field of prodrug design, further studies are needed, particularly in the early steps of the drug discovery, in order for prodrugs to accomplish the optimal efficacy and advance their role in contemporary pharmacotherapy.

## 4. Conclusions

This work presents a brief overview of the prodrug concept, classifications, recent developments, and clinical applications. We provide examples of potential uses and advantages of the contemporary prodrugs, such as overcoming solubility and permeability limitations, improving disease-targeting ability, overcoming extensive drug metabolism, and increasing treatment safety. Prodrugs are employed more and more as a tool to improve various drug characteristics, and their influence and development are expected to grow. Increasing knowledge of enzymes and membrane transporters in terms of structure, specificity toward different substrates, mechanism of action, and role can lead to the future development of successful targeted prodrug delivery. Prodrug design and synthesis are expected to change in the future through the use of different linkers/spacers, which can significantly improve drug delivery, targeting or controlling the rate of prodrug activation and release of the active parent drug. Besides, emerging computational methods play an important role in prodrug optimization and have the opportunity to significantly enhance the efficiency of the prodrug development process. Overall, prodrugs have become a vital part of the drug discovery toolbox, and their use is likely to further expand.

## Figures and Tables

**Table 1:** Prodrug structures and their therapeutic uses.

Prodrug	Structure	Therapeutic Uses
**Valacyclovir**	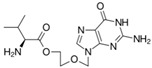	Herpesvirus [24,25]
**Tenofovir alafenamide**	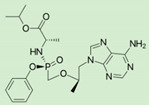	HIV/AIDS and chronic hepatitis B [47]
**Isavuconazonium sulfate**	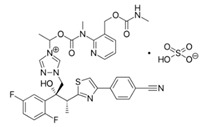	Invasive Aspergillosis/Mucormycosis [48,49]
**Gabapentin enacarbil**	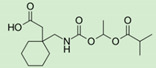	Restless leg syndrome, postherpetic neuralgia [50]
**Selexipag**	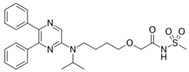	Pulmonary arterial hypertension [51]
**Prasugrel**	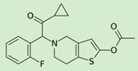	Prevention of thrombotic and cardiovascular events [52]
**NUC-1031**	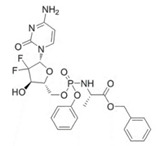	Advanced biliary tract cancer [53,54]
**OBI-3424**	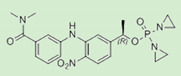	Relapsed/refractory T-cell acute lymphoblastic leukemia, hepatocellular carcinoma, and castrate-resistant prostate cancer [55,56,57]
**Baloxavir marboxil**	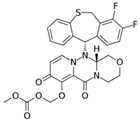	Influenza [58,59]
**Remdesivir**	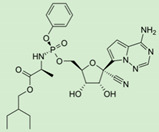	Coronavirus disease 2019 (COVID-19) in adults and adolescents with pneumonia requiring supplemental oxygen [60,61]
